# Adaptive SFC Management and Orchestration Based on DRL in Edge Intelligence for Computation Efficiency

**DOI:** 10.3390/s26134132

**Published:** 2026-06-30

**Authors:** Seyha Ros, Taikuong Iv, Intae Ryoo, Seokhoon Kim

**Affiliations:** 1 Department of Software Convergence, Soonchunhyang University, Asan 31538, Republic of Korea; rosseyha003@gmail.com (S.R.); taikuongiv@sch.ac.kr (T.I.); 2 Department of Computer Engineering, Kyung Hee University, Yongin-si 17104, Republic of Korea; itryoo@khu.ac.kr; 3 Department of Computer Software Engineering, Soonchunhyang University, Asan 31538, Republic of Korea

**Keywords:** computational efficiency, deep reinforcement learning, edge intelligence, network functions virtualization, service functions chaining

## Abstract

Network functions virtualization (NFV) is an emerging technology that enables flexible service deployment for supporting the Beyond 5G/6G network. NFV transforms physical network devices into virtual network functions (VNF) over Edge Computing capabilities, thereby facilitating the agility of network services and reducing management costs. To effectively monitor Internet of Things (IoT) network resources, service function chaining (SFC) is used for its virtualizations to ensure the multi-service requirements are sufficiently in capability, scalability, and flexibility for computation workloads alignments. However, to satisfy the resource availability requirements and efficiency under several conditions, SFC reconfiguration methods face the challenges in meeting significant latency requirement of delay-sensitive applications while reaching the importance of energy saving on orchestration timespan. In this paper, we propose task management-aware SFC and orchestrating schemes, namely GNN-PPO. In this framework, we utilize the Graph Neural Network (GNN), which relies on the message-passing neural network (MPNN), to capture all the abstraction of physical resource nodes and link capabilities over MEC node states. In particularly, GNN is divided construction into two phrases: (1) GNN represents nodes for all the Mobile edge computing (MEC) nodes, which have a global view on resources of computation and communicational capabilities that could serve as carriers; (2) VNFs are transferred into graph networks by using feature-extraction MPNN to manage each VIM that seeks an optimal and reliable analysis of traffic fluctuations. Lastly, Deep Reinforcement Learning (DRL) is used to embrace the network determination in policy strategy, which utilizes a Proximal Policy Gradient (PPO). On the other hand, we propose a novel network architecture based on PPO to perform the design for the optimization of resource utilization and facilitate energy consumption on MEC servers under diverse setting scenarios, which enables continuous policy enforcement for our system. With the experimental results, we compare our proposed solution with reference schemes in terms of rewards with learning rate and batch size, average request acceptance, SFC success, packet delivery, throughput, and resource utilization ratio that confirm the scheme’s scalability and practical suitability for IoT network deployment.

## 1. Introduction

**Figure 1:**
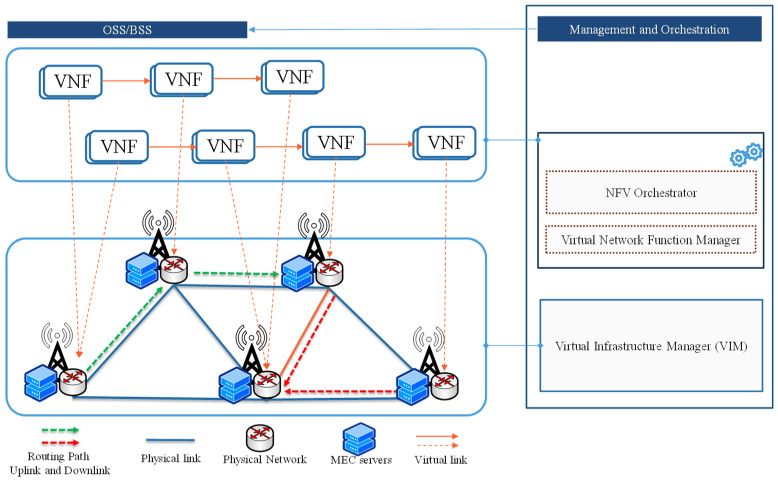
Overall SFC architecture deployed in MEC server for ensuring IoT networks.

With urban deployment of the internet of things (IoT) devices, increasing amounts of resource computation require both communication and computation capacities to fulfill in differentiation resource demands [1,2,3,4]. On the other hand, on-demand resources’ computing workload is trending into different B5G/6G network-specified types of service priorities and stringent quality of services (QoS) [5,6]. Meanwhile, the European Telecommunication Standards Institute (ETSI) proposed a network functions virtualization (NFV) framework to ensure the resource capabilities closest to end devices [7,8]. NFV has been used to tackle several challenges in terms of providing resource availability, dynamically and agilely consolidating functionality views over physical resources by adopting more declarative management and organization while addressing the end goals in providing more flexibility and easy integration perspectives [9]. On the other hand, multi-access edge computing (MEC) architecture provides the cutting-edge paradigms to deploy the running virtual machine (VM) for facilitating cloud computing capabilities to network edges, efficiently solving the traffic congestion bottleneck and ensuring the reliability service for terminal devices [10,11]. Especially, significant applications in IoT network are under handling within the MEC architecture. Figure 1 depicts the hierarchical resource alignment of diversities of service-based and network function on-demand. In this flow, management and orchestration (MANO) are set to arrange the resource pooling for handing the request from operation support system (OSS) and business support (BSS) [12,13,14,15].

However, with a heterogeneous number of IoT devices, traditional approaches are becoming unable to handle the massive traffic requests to satisfy the target requirements of low latency on computation workloads, resulting in resource-intensive, low-efficiency energy consumption in edge computing networks.

This limitation highlights the need for methods that explicitly overcome VNF allocation and SFC placement. Deep reinforcement learning (DRL) is leveraged to handle adaptive resource adjustment and mapping that provides a concise impact to MEC resource management [16,17,18]. Unlikely with traditional heuristic-based methods, this capability enables a DRL-based framework to learn from network interactions to optimize SFC placement and VNF resource allocation in real time. However, efficient orchestration remains challenging due to limited edge resources, unpredictable IoT workloads, and conflicting objectives such as QoS and reducing latency and energy consumption targets. Conventional static deployment strategies often fail to adapt to rapidly changing network conditions, as they are simultaneously required to support latency-sensitive and resource-intensive applications in real time. Consequently, the orchestration process must carefully balance multiple conflicting objectives, including minimizing end-to-end latency and energy consumption while maximizing resource utilization, service acceptance ratio, and operational efficiency.

In this paper, we utilize the Graph neural network (GNN) to embed with the hierarchical network in terms of network topology and resource nodes to capture long-term VNF-to-physical node dependency. On the other hand, message-passing neural network (MPNN) variance is employed to execute feature extraction from the physical network resource and SFC states [19,20]. Meanwhile, we design a two-level actor and critic structure with a Proximal Policy Gradient (PPO). The PPO is used to determine the policy for charging SFC deployment in the MEC server, enabling an adaptive allocation of optimal decisions, such as resources in fluctuating IoT networks. This integration with topology-aware learning mechanisms provides a promising direction toward autonomous and adaptive network orchestration for next-generation MEC-enabled IoT systems. Here is the brief of our main motivations and contributions:

- We utilize GNN to capture all spatial network topology by MPNN, including MEC nodes in long-range fluctuations. We design a topology-aware GNN-PPO framework that jointly addresses VNF allocation and mapping, as well as SFC placement by embedding MEC network structures, resource capacities, and service-chain dependencies into graph representations, enabling intelligent policy optimization for dynamic IoT workloads.
- We introduce a normalized, multi-objective System Utility formulation to mathematically reconcile the conflicting physical scales of energy consumption and processing latency. This prevents a reward explosion and ensures stable policy updates.
- We integrate GNN-based feature extraction with PPO-driven decision-making via a clipped surrogate objective, continuously optimizing VNF allocation and SFC routing while preventing the gradient instability observed in traditional heuristic and early-stage DRL baselines under fluctuating scenarios.
- We conduct extensive performance evaluations, demonstrating that the proposed GNN-PPO scheme consistently surpasses conventional DRL and heuristic baselines in service acceptance, convergence stability, throughput, and long-term computation efficiency for MEC-enabled SFC orchestration.

## 2. Related Work

Many research has investigated the optimization of resource management by using several techniques, such as service function chain offloading algorithm, VNF placement, and resource adjustment, which aim to alleviate network delays and maximize resource utilization to ensure computational capability. Enhancing the proactive precision for VNF resources is crucial in the MANO lifecycle, which involves the increase of energy and delays in orchestrating SFC forms for new templates. In this study, there are several investigations on existing works in terms of approach-based, algorithm-based, and deep learning-based variance in modeling.

By using heuristic algorithms to ensure resource optimization, Cai et al. utilized parallelized SFC techniques with an aim to increase accelerating SFCs. Their proposal utilized heuristic algorithms with distributed NFV architecture to implement SFC and develop parallelized serial SFCs based on the VNF dependency [21]. In [22], the author seeks to minimize the decision time and system’s latency for joint VNF deployment and migration by designing a multi-objective optimization problem. Their proposed algorithm utilizes a collaborative filtering-based fast delay-aware algorithm, which distributes information of deployed/migrated VNFs to assist the current VNF deployment in terms of reducing decision-making time. Thus, in summary, the heuristic algorithm depends on hand-crafted rules that cannot ensure the unseen traffic coming or topology fluctuation and get trapped in local optima.

Recent studies have explored DRL-based methods for dynamically adaptive SFC deployment, resource allocation, energy efficient, and VNF placement in dynamic edge networks environments. Zhang et al. introduced DeepSFC, a distributed self-learning method based on the Proximal Policy Optimization (PPO) algorithm. To overcome the challenges of sparse rewards in DRL, DeepSFC incorporates a curiosity-driven exploration module, enabling agents to make rapid, decentralized orchestration decisions that significantly improve network throughput under complex traffic patterns. While many approaches treat cost, latency, and reliability in isolation, recent frameworks attempt to optimize their complex interdependencies [23]. Xiao et al. introduced A2C-SFC, a two-stage DRL framework designed to meet Ultra-Reliable and Low-Latency Communication (URLLC) standards while minimizing Operational Expenditure (OPEX). In the first stage, A2C-SFC uses an Advantage Actor-Critic (A2C) algorithm integrated with a Karush-Kuhn-Tucker (KKT)-based adaptive resource preemption technique to allow active VNFs to preempt resources from unexecuted VNFs, thereby minimizing processing delays. In the second stage, it applies a reliability-aware redundancy management heuristic to optimally place redundant VNFs, achieving substantial OPEX reductions without violating strict latency and reliability constraints [24]. In [25], this paper proposed an approach for a SFC within an NFV-enabled network by addressing the complexity of both wired and wireless resources. Their proposed aim was to minimize E2E service latency, utilizing a Markov Decision Process (MDP) model of captured dynamics server capacity and radio interference. Additionally, they proposed a natural actor-critic deep reinforcement learning framework, which utilizes natural policy gradients to avoid local optima and improve training stability. On the other hand, to improve the energy-efficient offloading and resource allocation in the edge environment, ref. [26] explicitly presents a multi-user end-edge cloud orchestrated network that designs an optimal computation offloading and resource allocation strategy for computation-intensive and delay-sensitive tasks for particular edges on SBS or MBS, respectively. Additionally, ref. [27] utilizes an intelligent optimization strategy based on DRL for resource allocation strategies across a three-tier cloud-edge-end hierarchy directly relevant to the scalability and architectural positioning, as well as design cache replacement and task scheduling methods for arriving content requests. Their work utilizes asymmetrical control problems caused by a complex service and heterogenous network environment. This technique is to ensure content distribution for cloud-edge-end nodes. Ref. [28] considers integrating GNN and DRL agents, addressed to overcome complexity of network optimization by using MPNN to perform a feature extraction of the correlation between complex paths and links connection over network topology. However, this proposed method cannot reach QoS guarantees while optimizing resource and energy efficiency. In [29], the author introduces DRL-FJM, a joint mapping approach that simultaneously evaluates node and link resources to maximize the number of accepted service requests. The framework utilizes DRL and PPO to dynamically adapt to shifting network conditions and resource availability. Their technique provides a robust solution for managing the strict delay and capacity demands of modern applications like autonomous driving and IoT.

Through their proposed approaches and solutions for the problem, to push beyond the boundaries of these prior works, this paper proposes a substantive architectural leap. First, we introduce an MPNN variant that explicitly captures spatial link conditions (bandwidth, propagation delay) alongside node capacities, enabling topology-aware virtual routing. Second, we transition from standard DRL to a PPO architecture. This paper introduces a normalized, dynamically weighted System Utility formulation. This dimensionless objective resolves the mathematical instability caused by conflicting physical scales, allowing the PPO’s clipped surrogate objective to safely and consistently converge even under severe, fluctuating network congestion in B5G/6G MEC-enabled environments.

## 3. System Model and Problem Formulation

**Figure 2:**
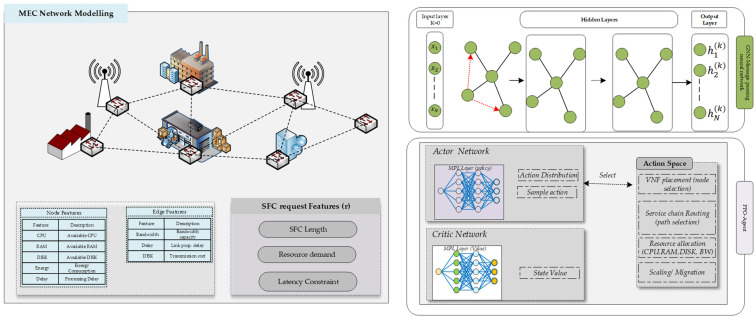
MEC-driven GNN-PPO in adaptively resource allocation and SFC placement.

This section, we consider the entire network infrastructure for problem formulation and representation of the VNF allocation and SFC placement over MEC servers. As shown, Figure 2 depicts the operational execution from the end-device tier to the control tier. All the VNFs associated with a request are proceeded with MEC servers. The workload on crucial tasks is primarily divided into categories based on network functionalities to achieve resource efficiency and energy consumption in the core network.

### 3.1. Architectural

MEC servers are deployed next to the end-device layer. Additionally, MEC has a control function that enables the SDN/NFV controller. In this context, SDN/NFV is centralized on traffic flow and resource management in terms of required QoS from the IoT request service. We divided it into two phrases: data flow and control flow, which are demonstrated below:

#### 3.1.1. Data Flow

The data flow is to initialize the connection between end-user and network devices by utilizing the communicating protocol and computing in form of the wireless and wired connection.

#### 3.1.2. Control Flow

Upon the arrival of a service request from IoT devices, all data are assumed to be fully offloaded from locally end devices to MEC servers instead. Due to local computing, delays on computing, energy consumption, and workload efficiency are hugely increased. Thus, MEC ensures the ability to overcome resource computing, proving to be reliable and stable in further needs.

### 3.2. System Models

We demonstrate the resource allocation and mapping into SFC templates to align resources that are demanding under MEC server computation and management. Typically, resource placement is the significant technique for ensuring the resource under controlling for differentiation in providing innovative solutions to the scalability problem of resource constraint.

#### 3.2.1. Network Model

The network model is modeled as directed graph $G=\left(V,E\right)$, where $V=\left\{v_{1},v_{2},\ldots v_{n}\right\}$ is the set number of MEC nodes. *E* is the set of physical links connecting between MEC. Let e,v∈V represent specific physical nodes and $e_{uv}\in E$ represent the physical link that connected node u to node v. The set of MEC servers possess a specific maximum resource capacity, denoted collectively as $C_{v}^{max}$, which encompassed computation resources such as CPU, RAM, and disk storage. Each physical link $e_{uv}$ has a maximum bandwidth capacity denoted by $C_{uv}^{bw}$. The physical network model hosts various VNFs to satisfy the diverse services required by IoT devices based on OSS/BSS demands. Our system aims to orchestrate these diverse services to strictly adhere to their Quality of Service (QoS) requirements. The unified notations used in our system model and formulations are provided in Table 1.

#### 3.2.2. Service Function Chaining Requests Model

In this system, diverse service requests are continuously generated by IoT devices and fully offloaded to the MEC servers for computation in each time slot t∈T. The set of service requests is denoted by Q, where each individual request q∈Q demands specific SFCs. An SFC request can be represented as a tuple, defining its operational and resource constraint that shows as $q=(F_{q},\;{cap}_{f}^{v},\;b_{f},{PT}_{f},{src}_{q},{dst}_{q})$, where $F_{q}\in F$ denotes the ordered sequence of VNFs that formulate the chain. The variable ${cap}_{f}^{v}$ denotes the required computation capacity to host a specific VNF f on node v, while $b_{f}$ denotes the required bandwidth capacity for the virtual links connecting the VNFs within the chain. Furthermore, ${PT}_{f}$ represents the maximum tolerable processing time for the network function to ensure delay-sensitive QoS requirements are met. Finally, ${src}_{q}$ and ${dst}_{q}$ define the ingress source and egress destination nodes for the traffic flow traversing the MEC network.

### 3.3. Problem Statement

Given the MECs with resource and service handling, the main objective is to ensure the service provider maximizes successful placement and orchestration while adhering to the associated constraints.

#### Constraint on Resource Allocation and Placement

The resource host capacity of proceeds over MECs is composed of utilizing currently available resources. Every MEC can be limited in terms of computation resources; for instance CPU, memory, and disk. The VNF is considered to manage and orchestrate following VIMs for the specific level and type of resource that are necessary to assign to a server on a specification workload. This constraint prevents the overwhelming of physical nodes.

(1) $$\sum_{f\in F}{cap}_{f}^{v}\;x_{f}^{v}\left(t\right)\leq C_{v}^{max},\forall v\in V,t\in T$$

In Equation (1), all the resources are ensured that they do not exceed their maximum available capacity $C_{v}^{max}$.

(2) $$\sum_{q\in Q}{∆}_{q}^{t}x_{f}^{v}\left(t\right)\leq {PT}_{f}$$

where ${∆}_{q}^{t}$ is the end-to-end latency by the service request q in timeslot t. Meanwhile, it can guarantee that resource requirements do not surpass the capacity at destination node following migration. Moreover, the processing load in every single node and bandwidth usage must not exceed in each MEC link, which remains through their respective capacity limits to ensure stable, feasible, and reliable network operation targets.

(3) $$\sum_{f\in F}b_{f}y_{f}^{uv}(t)\leq C_{uv}^{bw},\forall e\in E,t\in T$$

Equation (3) ensures the tolerable bandwidth demanded by $b_{f}$, with all virtual links mapping to a physical edge $e_{uv}$ that does not exceed the link’s maximum capacity $C_{uv}^{bw}.$

### 3.4. Delay Model

#### 3.4.1. Communication Delay

Communication considers the typically carrying packets within end-device and edge networks over the physical medium, which involves service delay criteria, e.g., queuing delays, transition delays, and propagation delays. We ensure they facilitate minimal delays on travers nodes from ingress to egress over chaining VNF nodes.

(4) $$D_{total}=D_{Process}+D_{queue}+D_{trans}+D_{prop}$$

where $D_{Process}$ is the node taken to examine the packet header and check for errors before determining the destination. $D_{queue}$ is in the prose as the dynamic buffer waiting time. This is highly dynamic and relies on network congestion levels. The time required to push all the packet’s bits onto the physical wire or wireless medium is determined by the packet length denote as L , and the transmission rate denotes as TR.

(5) $$D_{trans}=\frac{L}{TR}$$

$D_{trans}$ is the set of the period of time for the signal to physically travel from one end of the link to the other. Somehow, it depends on the distance and speed of the signal over the medium interface.

#### 3.4.2. Computation Delay on VNF

With computation delay on orchestrating VNFs, MECs are significantly considered for the resource to host virtual resource computing. This delay depends heavily on the computational workload required by the specific network function and the physical CPU resources allocated by the hosting node. To ensure that computation delay is minimized and kept within reasonable time limits for delay-sensitive applications, we mathematically formulate the processing delay for a given VNF as the ratio of its workload to the allocated server capacity.

(6) $$D_{comp}=\sum_{f\in F}\sum_{v\in V}x_{f}^{v}\left(t\right)\frac{W_{f}}{C_{v}^{cpu}}$$

#### 3.4.3. Optimistic Modelling in SFCs

To offer optimal performance in SFCs, we formulate the orchestration problem as a multi-objective optimization model. Rather than minimizing cost directly, we define a “System Utility” that inversely scales with latency and energy. The objective jointly maximizes this service utility while maximizing the service acceptance rate. The objective function is defined as:

(7) $$maxU=\sum_{t\in T}{\;⋋}_{lat}\left(t\right)\left(1-\frac{{\;D}_{t}}{{\;D}_{MAX}}\right)+{\;⋋}_{ene}\left(t\right)\left(1-\frac{{\;E}_{t}}{{\;E}_{MAX}}\right)+{\;⋋}_{acc}A_{t}$$

where ${\;D}_{t}$ and ${\;E}_{t}$ denote the latency and energy consumption at time slot-t, respectively, and ${\;D}_{MAX}$, $E_{MAX}$ are normalization constants. $A_{t}\in \left\{0,1\right\}$ represents whether the SFC request is successfully accepted. The coefficients ${\;⋋}_{lat}\left(t\right)$, ${\;⋋}_{ene}\left(t\right)$, and ${\;⋋}_{acc}\left(t\right)$ are weighting factors that dynamically balance the optimization objectives, subject to: (1)–(3).

## 4. Methodology

To address the placement strategy, we utilize actor–critic mechanism for defining VNF allocation and SFC placement by adopting Markov decision process (MDP) framework. Hence, the main comprises six elements: environment, agent, state space, action space, reward, and design policy enforcement.

Agent: within the agent, there consisted of two components: Actor and Critic. Actor is to set the interaction directly with network environments, which performs selecting action relying on current policy $\pi_{\theta }\left(a_{t}|s_{t}\right)$, which means choosing the probability an action $a_{t}$ in state $s_{t}$. Meanwhile, critic evaluates the perform strategy by learning a value function $V_{\omega }\left(s_{t}\right)$ and output of the estimated value by the discounted cumulative reward $\;s_{t+1}$.

### 4.1. Hierarchical DRL Based on Graph-Passing Neural Network Variance

With capacities of network states being crucial in capturing the complex topology and dynamic resource fluctuation distribution of the MEC nodes, we incorporate a GNN based on MPNN variance. In this work, the physical network is represented as directed graph. On each node, v is associated with feature vector:

(8) $$x_{v}=\left[CPU,RAM,DISK,\;{Energy}_{v},\;{Delay}_{v}\right]$$

where $C_{uv}^{bw}$ and ${PD}_{uv}$ are available bandwidth and processing delay at node v, respectively. Similarly, each edge $e_{uv}$ is feature vector to capture the spatial dependencies and propagation delay of the link, respectively.

To capture the spatial dependencies and resource interactions among nodes, the GNN adopts a message-passing mechanism. At each layer k, node v receives messages from its neighboring nodes $u\in N\left(v\right),$ where $N\left(v\right)$ denotes the set of adjacency nodes. Additionally, $m_{v}^{\left(k\right)}$ represents the aggregated message for node v at layer κ, and $\phi \left(\cdot \right)$ is a learnable message function implemented using a multilayer perceptron (MLP). This function integrates both the neighbor node embedding $h_{u}^{\left(k-1\right)}$ and the corresponding edge features ${\;e}_{uv}$, allowing the model to jointly consider resource states and link conditions during information propagation.

(9) $${\;e}_{uv}=\left[C_{uv}^{bw},{PD}_{uv}\right]$$

(10) $$m_{v}^{\left(k\right)}=\sum_{u\in N\left(v\right)}\emptyset \left(h_{u}^{\left(k-1\right)},{\;e}_{uv}\right)$$

By following MPNN, each node updates its embedding by combining its previous representation with the aggregated neighborhood information. The update rule at layer k is given by:

(11) $$h_{v}^{\left(k\right)}=\sigma \left(W^{\left(k\right)}\cdot \left[h_{v}^{\left(k-1\right)}\left\|m_{v}^{\left(k\right)}\right.\right]+b^{\left(k\right)}\right)$$

Here, $h_{v}^{\left(k\right)}$ is the embedding of nodes v at layer  κ, and $W^{\left(k\right)}\;$ and $b^{\left(k\right)}$ are trainable parameters. The function $\sigma \left(\cdot \right)$ denotes a nonlinear activation function such as ReLU. Finally, a graph-level embedding is obtained to capture all states of MEC network. It is achieved using a permutation-invariant readout function, which is defined as:

(12) $$z_{G}=\frac{1}{\left|V\right|}\sum_{v\in V}h_{v}^{\left(k\right)}$$

#### 4.1.1. State Space

The state encapsulates VNF resource and requested SFC information from GNN execution, formally defined as:

- $R_{coord}^{t}$: set of the coordination of resource (CPU, RAM, Disk) in MEC server to handle computation n tasks.
- $z_{G}$: the global graph embedding extracted from the readout layer the GNN, capture the current network topology.
- $U_{Max}$: the set of upper-bound resource utilization in MEC node-m at timeslot-t.
- $B_{uv}^{t}$: state of total bandwidth.
- $T_{s}$: a tuple representation of processing task divides into resource consumption on computation VNF, energy, and time spent.
- req: the specific SFC request feature, such as SFC length, resource demand, and latency constraint.

(13) $$S_{t}=\left\{{z_{G},R}_{coord}^{t},U_{Max},B_{uv}^{t},T_{s},req\right\}$$

By leveraging this GNN-based state representation, the DRL agent can efficiently and adaptively manage the resource-awareness information more concisely of the network, enabling decision for VNF allocation and SFC placement under dynamic network workloads.

#### 4.1.2. Action Space

With action selection, selection placement decision of SFC and VNF allocation is:

(14) $$a_{t}=\left\{x_{f}^{v}\left(t\right),y_{f}^{uv}\left(t\right),\;{alloc}_{f}^{v}\right\}$$

Here, $x_{f}^{v}\left(t\right)\in \left\{0,1\right\}$ sets the decision to place VNF on physical node, while $y_{f}^{uv}\left(t\right)\in \left\{0,1\right\}$ selects the routing decision on link placement between nodes. Lastly, ${alloc}_{f}^{v}$ depicts the decision on service function request for allocating resource service function types.

#### 4.1.3. Reward

During the latency-aware SFC placement and VNF allocation phase, our objective aims to minimize both the MEC server latency for initial VNF instance and the energy for execution. Thus, the reward function is as follows:

(15) $${\;r}_{t}={\;⋋}_{lat}\left(t\right)\left(1-\frac{{\;D}_{t}}{{\;D}_{MAX}}\right)+{\;⋋}_{ene}\left(t\right)\left(1-\frac{{\;E}_{t}}{{\;E}_{MAX}}\right)+{\;⋋}_{acc}A_{t}-P_{t}$$

where $P_{t}$ is the penalty applied for constraint violation. ${⋋}_{lat}$ and ${⋋}_{Ene}$ are time-varying weights ${(⋋}_{lat}+{⋋}_{ene}=1)$ that adapt based on current conditions. When the network load is high or delay-sensitive service requests are prioritized, ${⋋}_{lat}\left(t\right)\;$ is increased to focus on reducing latency. Conversely, under lower loads or when energy conservation is critical, $\;{⋋}_{Ene}\left(t\right)$ is emphasized to minimize energy consumption.

### 4.2. Hierarchical Reinfocement Learning Based on PPO Framework

PPO is widely used for its efficiency and significant performance combined with RL to enhance learning policy. We employ a PPO-based actor-critic architecture consisting of: (1), actor network $\;\pi_{\theta }\left(a|s\right)$ chooses a probability distribution over action, and (2), critic network $V_{\theta }\left(s\right)$ estimates the state value function. The policies have identified clipped surrogate objective functions as in Equation (16). Otherwise, in (17), generalized advantage estimation is utilized for calculation in ${\hat{A}}_{t}$ advantage estimate.

(16) $$L^{CLIP}(\theta )={\hat{E}}_{t}[\min\left(p_{t}\left(\theta \right){\hat{A}}_{t},clip\left(r_{t}\left(\theta \right),1-\varepsilon ,1+\varepsilon \right){\hat{A}}_{t}\right]$$

(17) $${\hat{A}}_{t}=\sum_{l=0}^{\infty }{\left(\gamma \;⋋\right)}^{l}\delta_{t+l}$$

(18) $$\delta_{t}=r_{t}+\gamma V\left(s_{t+1}\right)-V\left(s_{t}\right)$$

(19) $$\theta \leftarrow \theta +\alpha_{\theta }\frac{L^{CLIP}\left(\theta \right)}{\partial \theta }$$

### 4.3. Algorithms Design

**Figure 3:**
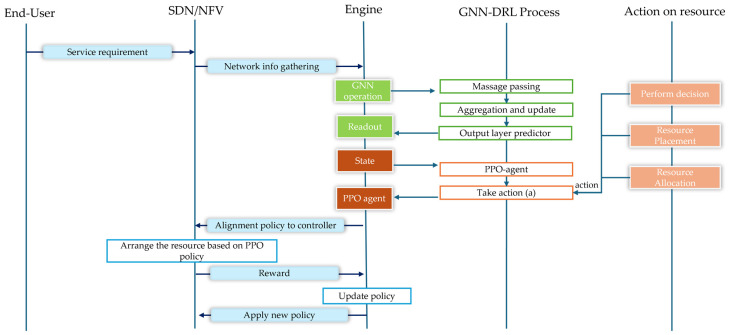
State procedure PPO-enable SDN/NFV for resource orchestration.

Our scheme is to enhance the network resource on executing the management for SFC orchestrations. As shown, Figure 3 depicts the state procedure of initial network components. This objective aims to fulfill reliability requirement handling while minimizing the latency on orchestration and energy consumption within centric. The hierarchical set between GNN is incorporated with DRL to execute policy enforcement in efficient resource management and control the packets from end user on resource demand in SDN/NFV controller.

### 4.4. Joint Adaptively Resource Orchestration Based on GNN-PPO Framework

To adaptively orchestrate, GNN-PPO is joined for decision-making for handling resource orchestration and management over MEC servers. Algorithm 1 depicts an execution as a closed-loop optimization cycle to achieve joint VNF allocation and SFC orchestration. At each time step, the current network topology $G_{t}$ and incoming service request are fed into a K-layer MPNN to extract topology-aware spatial and resource dependencies. Through k iterations of recursive message passing, the MPNN aggregates localized neighborhood attributes to generate refined node embeddings $h_{v}^{\left(k\right)}$. A permutation-invariant readout layer subsequently condenses into a global graph embedding. This graph representation is concatenated with immediate SFC constraints to construct the composite environment state vector. Leveraging this comprehensive state representation, the PPO Actor network yields an orchestration decision $a_{t}$ ~ π ($a_{t}|s_{t}$) defining specific VNF placement locations and traffic routing paths. Following execution within the MEC environment where processing, bandwidth, and latency constraints are verified, the system computes a scalar reward reflecting service acceptance and resource conservation Trajectory transitions are collected in an on-policy rollout buffer D. Once the rollout buffer reaches the specified batch size threshold, it triggers a joint network update. Here, the Generalized Advantage Estimates (GAE) are computed via the Critic network to optimize the Actor policy and GNN parameters via the clipped PPO objective while minimizing the Critic’s Mean Squared Error (MSE) value loss.
**Algorithm 1:** Adaptively joint VNF allocation and SFC placement-based GNN-PPO framework 
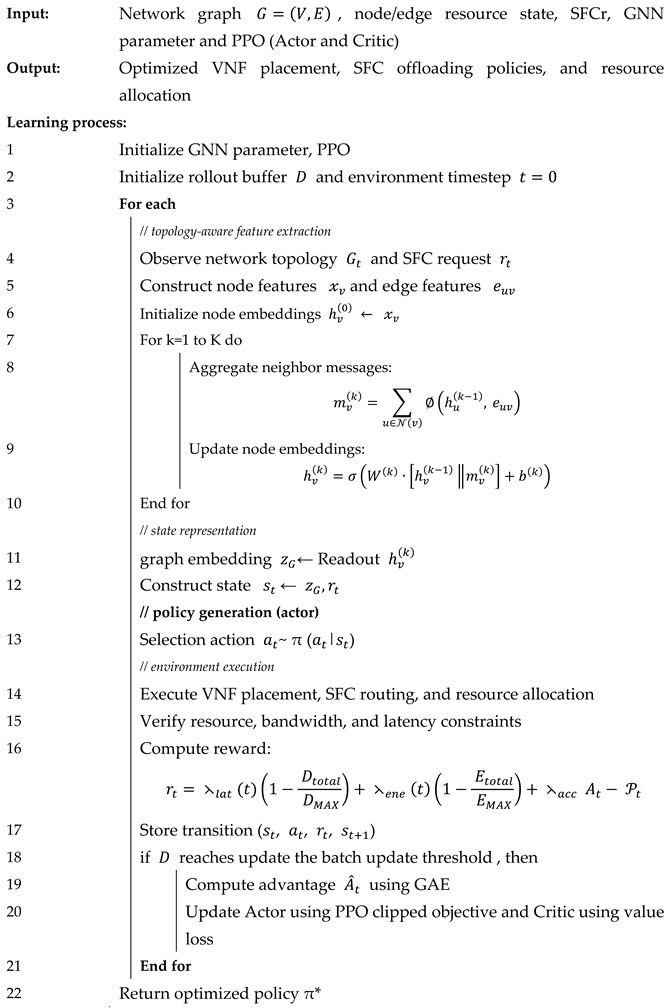
 

## 5. Performance Evaluation

To evaluate the GNN-PPO framework, we simulate the dynamic MEC environment using the python program, which utilizes the NetworkX library to create network topologies. We employ PyTorch for the Deep Learning model and Gymnasium for DRL, and the vary network conditions are considered in three types. Additionally, OASIS TOSCA NVF is utilized to create the concept of service temple for topology template in terms of network function deployment in this study [30,31]. To accurately reflect realistic B5G/6G edge computing scenarios, the network topology is instantiated as a partially meshed graph of five MEC servers with an edge connection probability of 0.6, representing dispersed edge density. Service requests from the 100 IoT devices are dynamically generated following a Poisson arrival process with a rate of (10, 50) requests per timeslot. Each request demands an SFC consisting of two to five VNFs drawn uniformly from five distinct VNF types (e.g., Firewall, NAT, IDS).

Furthermore, to calculate the energy consumption metric for the System Utility objective, we adopt a standard linear-server energy consumption model. The energy consumed by MEC server v at time t  is calculated as $E_{v}=P_{idle}+\left(P_{max}-P_{idle}\right)\times U_{v}\left(t\right),$ where $P_{idle}$ and $P_{max}$ are the idle and peak power consumption, and $U_{v}\left(t\right)$ denotes the current CPU utilization ratio of the server [32]. The complete granular configurations for the network resources, service, and DRL hyperparameters are summarized in Table 2.

### 5.1. Comparison of Propose and Reference Schemes

In this study, we validate our performance method compared with the four baselines.

- DQN utilizes a Deep Q-Network to learn control policies from network states, but its performance declines in complex topologies, making it more suitable for lightweight network environments.
- Load-balancing focuses on distributing workloads evenly across available resources by selecting target networks and assigning VNFs to SFCs based on priority. While it improves utilization and prevents overload, it struggles to adapt to strict or dynamic VNF priority constraints.
- Greedy reflects a traditional SDN/NFV-based MANO strategy for MEC resource management, relying on centralized topology-aware optimization, traffic conditions, and service policies. It performs adequately for simple, non-complex applications but lacks intelligence for dynamic large-scale orchestration.
- Static-on resource uses stochastic MEC service selection from ingress nodes through predefined VNF instances and routing paths for each IoT request, offering simple service chaining but limited adaptability and optimization efficiency.

### 5.2. Results and Discussions

In this section, we present the results for the proposed GNN-PPO and reference schemes, namely DQN, Load-balance, Greedy, and Static. In this stage, we perform our proposal explicitly in terms of learning rate and batch size.

**Figure 4:**
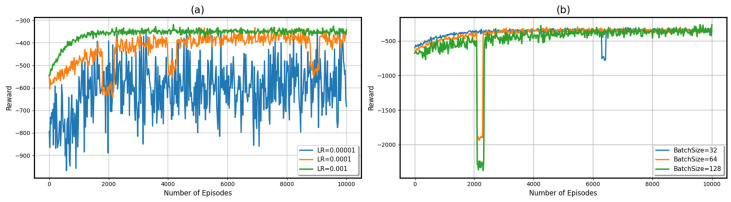
Performance metrics on rewards of (**a**) learning rate and (**b**) batch size.

In Figure 4a, the influence of learning rate (LR) on the convergence behavior of the proposed GNN-PPO framework is evaluated under three configurations (0.00001, 0.0001, and 0.001). During the initial training stage, all configurations exhibit highly negative rewards, indicating inefficient policy exploration as the GNN encoder and PPO policy network are still learning to interpret topological states, resource availability, and SFC demands within the MEC-enabled IoT environment. At this phase, GNN has not yet fully captured the structural dependencies between network nodes, while PPO’s actor–critic policy remains unstable due to random action selection. As training progresses, LR (0.00001) demonstrates extremely slow adaptation, with severe reward oscillations persisting across episodes, suggesting that the PPO optimizer updates the GNN-extracted embeddings too conservatively, thereby limiting effective policy refinement for VNF placement and resource orchestration. LR (0.0001) provides improved convergence by enabling more balanced policy gradient updates, allowing the PPO agent to better utilize GNN-based topology-aware state embeddings for adaptive resource scheduling. However, moderate fluctuations remain, indicating slower responsiveness to dynamic workload variations. In contrast, LR (0.001) achieves the fastest and most stable reward convergence, rapidly improving within early episodes and consistently maintaining the highest reward levels. Figure 4b illustrates the effect of batch size on the training efficiency of the proposed GNN-PPO model, where BatchSizes (32, 64, and 128) are compared. In early episodes, all batch sizes begin with substantially negative rewards due to incomplete policy awareness of network topology and computational resource distribution. However, because the proposed model integrates GNN-based message passing to encode node connectivity, link capacity, and distributed MEC resource states, PPO gradually improves decision quality as batch updates refine both actor and critic networks. BatchSize (32) initially converges faster because smaller batches enable more frequent PPO parameter updates, allowing the GNN-PPO agent to quickly adapt to short-term resource orchestration patterns. Nevertheless, this configuration also suffers from noticeable reward instability, including sudden performance drops, indicating that overly frequent updates may amplify variance in policy gradients and reduce long-term stability. BatchSize at 64 demonstrates the most effective balance, achieving robust convergence with fewer severe oscillations. This suggests that moderate batch sizes allow the GNN to generate sufficiently informative topology embeddings while PPO benefits from stable gradient estimation, producing consistent optimization of SFC placement, node utilization, and energy-aware scheduling. BatchSize at 128, although initially slower and exhibiting deeper instability, eventually stabilizes as larger batch aggregation improves PPO’s gradient consistency; however, delayed responsiveness reduces adaptation speed to dynamic MEC workload changes.

**Figure 5:**
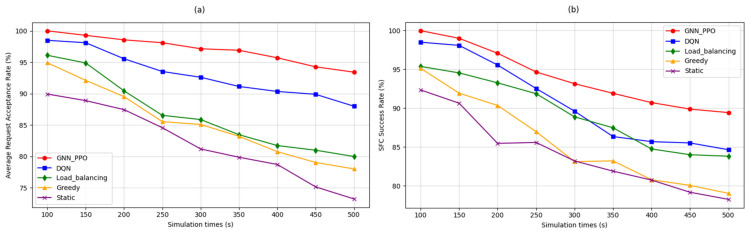
Performance metrics on (**a**) average request acceptance and (**b**) SFC success rate.

The results are demonstrated in Figure 5a,b for average request acceptance and SFC success rate, respectively, where network states are configured consecutively by setting different network conditions, congestion levels, and application tasks. Figure 5a shows the average SFC request acceptance ratio as service demand increases from 100 to 500 requests over simulation time. All methods experience declining acceptance rates due to higher resource contention and network saturation; however, the proposed method consistently outperforms all baseline schemes across all traffic levels. At 100 requests, GNN-PPO achieves nearly perfect acceptance (99.998%), surpassing DQN (98.49%), Load-balancing (96.11%), Greedy (94.92%), and Static (89.93%). Even under heavy workload at 500 requests, the proposed method maintains 93.42% acceptance, significantly higher than DQN (87.99%) and conventional approaches. Meanwhile, Figure 5b demonstrates a consistently better performance than DQN, Load-balancing, Greedy, and Static approaches across all simulation times. At 100 simulation time, GNN-PPO achieves the highest success ratio at 99.91%, demonstrating superior topology-aware SFC placement and resource orchestration through the integration of GNN-based network representation and PPO policy optimization. Although all methods decline as simulation time increases due to higher resource contention and network complexity, GNN-PPO maintains the strongest performance, retaining 88.42% at 500 simulation time, compared to DQN (82.17%), Load-balancing (79.99%), Greedy (78.01%), and Static (73.23%).

**Figure 6:**
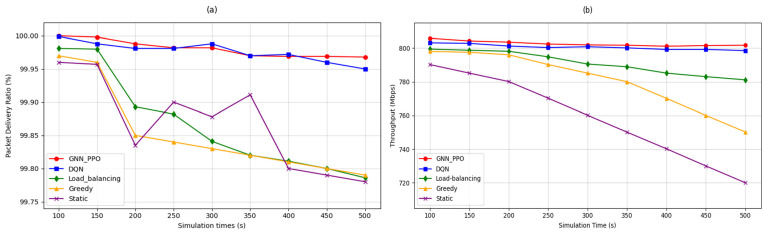
Performance metrics of (**a**) packet delivery and (**b**) throughput.

With the results presented in Figure 6a, GNN-PPO achieves the highest PDR at 99.98% during the initial stage and maintains superior stability with 99.96% at 500 simulation time, demonstrating more reliable packet transmission under dynamic MEC conditions. While all methods experience slight degradation as network complexity increases, GNN-PPO shows the smallest performance decline due to its integration of GNN-based topology awareness and PPO-driven adaptive policy optimization. Figure 6b shows that the proposed GNN-PPO method achieves the highest and most stable performance, with throughput decreasing only slightly from 805.8 Mb/s to 801.7 Mb/s (drop of 4.1 Mb/s), indicating strong adaptability and efficient resource orchestration under dynamic network conditions. DQN performs similarly, but it experiences a slightly larger decline (4.9 Mb/s), while Load Balancing, Greedy, and Static show significantly greater throughput degradation due to less adaptive decision-making.

**Figure 7:**
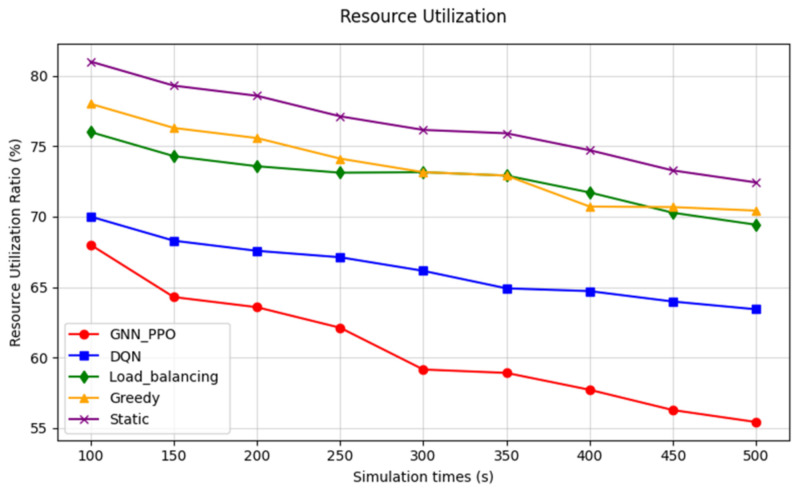
Resource utilization ratio (note: lower values indicate better performance via reduced resource overhead).

Figure 7 depicts the GNN-PPO framework consistently minimizing resource consumption across all simulation intervals, demonstrating superior efficiency in VNF placement, SFC orchestration, and adaptive resource allocation. Specifically, GNN-PPO reduces resource utilization from an initial 67.99% to 55.42% by simulation time 500, highlighting its capacity to intelligently allocate computational and network assets with minimal overhead. Conversely, DQN maintains higher utilization levels, declining only to 63.42% (from 69.99%). Traditional baselines Load Balancing, Greedy, and Static exhibit the poorest performance, ending at 69.42%, 70.42%, and 72.42%, respectively, due to their rigid orchestration.

These outcomes confirm that the GNN successfully captures topology-aware resource dependencies, while PPO dynamically optimizes VNF placement and SFC offloading, thereby maximizing scalability, congestion handling, and resource utilization. In contrast, DQN shows moderate degradation, while conventional baselines (Load-balancing, Greedy, and Static) decline sharply due to limited adaptability.

## 6. Conclusions

In this study, we proposed a novelty approach for enhancing VNF placement and SFC orchestration in MEC server environment through integration of GNN incorporated with the PPO framework. The proposed method framework demonstrated its effectiveness by reducing the time of orchestrating resource intensive and energy consumption in dynamic resource workload over MEC scenarios, while GNN-PPO provides a significantly better solution for fast response capacities on resource-constraint edge devices. By integrating a message passing neural network into a PPO (actor, critic) architecture, in this phrase, MPNN captures the state of SFC features and network topologies, thereby achieving coupled decision-making for node mapping. Otherwise, PPO determines the policy for charging SFC deployment in the MEC server, enabling adaptive allocation of optimal decisions for resource instances. Our simulation result outperforms the reference scheme in terms of reducing latency and energy consumption targets in complex task scenarios.

## Figures and Tables

**Table 1:** Notation system and variables.

Notations	Description
Network Resources	
$G=\left(V,E\right)$	The graph of physical network G where decouples the set of node and link set E
u,v∈V	Physical MEC servers/nodes
$e_{uv}\in E$	Physical link connecting node u to node v
N	Set of number IoT devices N=1,2,…,n, ∀n∈N
T	Timeslot−t
Resource capacities and SFC Requests	
$C_{v}^{CPU}$	Allocated CPU processing rate (e.g., cycles per second) of MEC node v
$C_{v}^{max}$	Maximum available capacity (CPU, RAM, Disk) of MECs node v
$C_{uv}^{bw}$	Maximum bandwidth capacity of physical link $e_{uv}$
${cap}_{f}^{v}$	Required computation capacity to host a specific VNF f on node v
$b_{f}$	Required bandwidth capacity for service request over a virtual link
${PT}_{f}$	Maximum tolerable processing time for network function f
${\;D}_{MAX},{\;E}_{MAX}$	Normalization constants for maximum system delay and energy
$W_{f}$	Required computational workload to completely process network function f
*F*, Q	Set of available Network function (f∈F) and service requests $\left(q\in Q\right)$
Binary Variables	
$x_{f}^{v}\left(t\right)$	Binary variable indicating whether service request that deploy MECs node at time-t, otherwise
$y_{f}^{uv}\left(t\right)$	Binary variable indicating whether virtual link between node at time-t, otherwise
${\;⋋}_{lat},{\;⋋}_{ene},{\;⋋}_{acc}$	Dynamic weight coefficient for latency, energy, and acceptance optimization objectives.

**Table 2:** Parameters setting on simulation and hyperparameter.

Parameters	Value
Hosting network infrastructure	Intel(R) i7-12700 CPU @ 2.10 GHz, Ram 32 GB @ 3200 Mhz
Number of IoT (end-devices)	100
Number of MECs Bandwidth on link	5
	Random set (100–1000 Mbps)
Link propagation delay	(1–5 ms)
Topology and edge density	Partially meshed graph, edge connection probability *p* = 0.6
Service Configuration	
VNF	VNF 5 types
Request arrival rate	Poisson distribution (10–50) req/timeslot
SFC type of length	(2–5)
Virtual link demand/Flow rate	(64 Kbps–4 Mbps)
The configuration of DRL models	
Learning rate	Value subset (0.00001, 0.0001, 0.001)
Hidden size	Value subset (32, 64, 128)
Discount factor	0.95
GAE trace-decay	0.95
PPO clipping	0.2
Buffer size	1 × 10^5^
Batch size	Value subset (32, 64, 128)
Number of episodes	10,000
Python platform	PyTorch v2.11.0

## Data Availability

Derived data supporting the findings of this study are available from the corresponding author on request.
